# Attention-Seeking Displays

**DOI:** 10.1371/journal.pone.0135379

**Published:** 2015-08-19

**Authors:** Szabolcs Számadó

**Affiliations:** MTA-ELTE Theoretical Biology and Evolutionary Ecology Research Group, Eötvös Loránd University, Department of Plant Taxonomy and Ecology, Budapest, Hungary; University of Western Australia, AUSTRALIA

## Abstract

Animal communication abounds with extravagant displays. These signals are usually interpreted as costly signals of quality. However, there is another important function for these signals: to call the attention of the receiver to the signaller. While there is abundant empirical evidence to show the importance of this stage, it is not yet incorporated into standard signalling theory. Here I investigate a general model of signalling - based on a basic action-response game - that incorporates this searching stage. I show that giving attention-seeking displays and searching for them can be an ESS. This is a very general result and holds regardless whether only the high quality signallers or both high and low types give them. These signals need not be costly at the equilibrium and they need not be honest signals of any quality, as their function is not to signal quality but simply to call the attention of the potential receivers. These kind of displays are probably more common than their current weight in the literature would suggest.

## Background

The potential mechanisms behind reliable signalling are of great interest and importance in biology, in economics and in the social sciences [1,2]. This process is usually modelled as a signalling game, discrete or continuous, between a signaller and a receiver [3,4,5,6,7,8] see [9] for a review. These models have been successful in describing the conditions for honest signalling as well as other types of equilibria from pooling to mixed cheating [10,11,12]. Almost all of these signalling models investigate a pair-wise interaction of a signaller and a receiver and look for an evolutionarily stable pair of signaller and receiver strategies [6,8].

However, real life situations could be more complicated than the above simple scenario. For example, receivers often have to find signallers. Signallers may help this process by giving signals that are easy to locate. This is not a new idea; Richards [13] had already proposed the separation of bird song into an alerting and a message component. He argues that “the alerting component must be highly detectable; this requires low degradation during transmission, and high contrast to extraneous perturbations.” (pp. 227) He investigated the song of rufus-sided towhees (*Pipilo erythropthalmus*) and found that the introductory trill serves this function. He also argues that this kind of functional separation can be found in many more bird species (pp. 224) and in mammals (mangabey, *Cercocebus albigena*) as well. This kind of “alert signals” have been found in other species too, like the introductory push up displays of *Anolis* lizard (*Anolis gundlachi*, [14]), or the introductory tail-flick of Jacky dragons (*Amphibolurus muricatus*, [15]).

There are plenty of empirical studies that show that the initial attraction and the final mate choice can be influenced by different parts of multicomponent displays. Examples include: spotted bowerbird (*Chlamydera maculata*) nests where white bones serve as long distance displays to attract females, yet it is the glass decoration that determines final mate choice [16]; satin bowerbird (*Ptilonochyncus violaceus*) mating display where the female visitation rates depend on vocal display rate yet mating success depends on the size of the male and on the quality of decorations [17]; sage grouse (*Centrocercus urophasianus*) mating display where female visitation rates depend on the signal interval yet mating success depends on the display rate [18]; ring-necked pheasant (*Phasianus colchicus*) mating display where original mate attraction depends on feeding courtship behaviour and copulation acceptance depends on lateral-display [19]. Last but not least, the mating display of the peacock (*Pavo cristatus*) where different traits of the tail ornament serve different functions [20]: the maximal change in colour contrast is used by females to find males at long distance, whereas the brightness of the eyespots is used to assess males at short range. One conclusion of these studies is that signals that function to attract are highly conspicuous and detectable over long distances.

While this attention-seeking stage is clearly demonstrated in nature and it is argued to be an important stage in mate choice [16], this stage has never been explicitly modelled in signalling games. Here I investigate a general model of signalling that incorporates this searching stage; it is based on the widely used action-response game [4,5,6,9]. Action-response games are used to describe asymmetric signalling situations where the signaller is in a possession of a resource. These games can be used to model situations with and without relatedness, for example parent-offspring conflict or mate choice, see [9] for review. I call signals that are designed to help this search “*attention-seeking displays*” (ASD) as their function is not necessarily to signal quality but simply to call the attention of potential receivers. I prefer this term compared to “alert signals”, a term used in the previous studies [14, 15], because alertness can refer to external threats as well, such as predators.

Note, it is important to make a distinction between the so called ‘alert displays’ where the function of the displays is to grab the attention of an already present receiver (like the introductory push up displays of *Anolis* lizard *Anolis gundlachi*, [14]) and between those displays that designed to help the localization of the receiver (e.g. spotted bowerbird *Chlamydera maculata* [16]). These later displays are expected to operate from a larger distance than the previous ones. Accordingly, the model considers a situation where receivers have to search for signallers and signallers have a choice to produce or not to produce displays that help the receiver in their search.

## Methods

The model is based on a general action-response game [4,5,6]. This type of signalling game is used to describe situations where receivers control a (non-divisible) resource that the signallers wish to obtain, like parent-offspring communication or mate choice. Aggressive communication where the players compete for the resource has a different structure and it is modelled by a different set of games (Enquist, 1995, Hurd & Enquist, 2005). The model is a two-player game: one player is the receiver who has a non-divisible resource at her disposal; the other player is the signaller who wants to get the resource. Signallers can be high or low types, this quality is hidden from the receivers. The signallers are aware of their own quality and they may or may not give an honest signal that reveals their quality to the receivers. Receivers are interested in sharing the resource with the high quality signallers but not with the low quality ones. It is assumed that the signaller and the receiver are not positioned close to each other at the beginning of the game, thus receivers have to search for signallers. This search is costly and it can be facilitated by giving an attention-seeking display by the signallers that has a cost but increases the probability of receivers discovering the signaller (*p*). In the absence of ASD the receiver still can find the signallers with a decreased probability (*q*), where *q*<*p*≤1. The receiver can choose not to search for ASDs; in this case the receiver’s chance to find a signaller is diminished (0≤*α*<1) independently of the decision of the signaller to give or not to give ASD. If *α* = 0 then receivers cannot find signallers without searching for ASDs, if *α* = 1 then receivers can perfectly locate signallers without searching for ASDs (in which case there would be no use for ASDs). After the receiver has searched and found a signaller, the signaller now uses a second display to signal its quality to the receiver. Based on this quality signal, the receiver decides to transfer or withhold the resource to the signaller. In other words, ASDs and search for them only influence the probability of finding the signaller, but they do not have an influence on the receiver's decision to transfer resources to the signaller.

One has to note that the same model described above is suitable to model alert displays as well, where the signaller and the receiver are already at the same location but the receiver is engaged in some other activity. Giving an alert display in this situation increases the chance that the receiver is attentive to the signals displayed by the signaller. Thus the probability of being attentive will be increased from *q* to *p* (*q*< *p* ≤ 1), where *q* reflects the probability that the receiver disengages with the other activity and notes the receiver, in absence of any ASD; similarly, *p* reflects the probability that the receiver takes notice of the receiver that uses an ASD. Of course, signals that serve to attract receivers from a long range are probably costlier than short range attention grabbing displays, but this cost parameter in the model can be easily adjusted.

Note that both interpretations assume some simplifications (i.e. receiver is not present vs. receiver is present but engaged in some other activity). In the first case it is assumed that once the signaller is found the attention of the receiver is given to the signaller. In the second case it is assumed that the signaller and the receiver are already at the same location. A more complex model (a three-step one) would be required to model the whole process. For the sake of simplicity we will investigate a two-stage version with a “search” terminology and with a quality signalling stage.

The technical description of the model is as follows. We will use the most general notation (Hurd, 1995; Számadó 1999) without making any specific assumptions about the shape of functions used in the model (however, in order to make the model more acessible I will give four different parametrizations after the general results, using examples from the literature). Note that in some action-response games, most notably the Sir Philip Sidney game [4] and its derived models explicit (linear) functions assumed, which makes the notation more compact but less open to general interpretation. The receivers’ fitness (*F*_*r*_) is the sum of the value of the receiver’s action (*W*) minus the cost of search (*C*_*r*_). The receiver’s response depends both on the signaller’s quality (*a*), which can be high (*H*) or low (*L*) and on the receiver’s response (*z*), which can be up (*U*): to give the resource, or down (*D*): not to give the resource. The cost of search depends on the receiver’s behaviour (*b*_*r*_), which can be to search for ASDs (*SA*) or not to search (*NS*). The signaller’s fitness (*F*_*s*_) is the sum of the value of the receiver’s response (*V*), minus the cost of quality signalling (*C*) and the cost of the attention-seeking displays (*C*_*a*_). The value of the receiver’s response (*V*) both depends on the quality of the signaller and on the receiver’s response; *C* depends on the quality of the signaller too and on the signaller’s behaviour (*b*_*s*_), which can be to signal (*S*) or not to signal (*N*); and finally *C*_*a*_ depends on the same variables as *C*, where the behaviour (*b*_*as*_) represents an independent decision to signal (*S*) or not to signal (*N*) in the context of attention-seeking. Accordingly, *F*_*r*_ and *F*_*s*_ can be written up respectively as follows:

$$F_{r}=W(a,z)-C_{r}(b_{r})$$ (1)

$$F_{s}=V(a,z)-C(a,b_{s})-C_{a}(a,b_{as})$$ (2)

The fitness of each player can be influenced by the other player outside of the context of signalling (*r*) which can mean, for instance, that they are relatives, or they can help each other some other way (e.g. they belong to the same group, see [4]). Note, that with the help of *r* it is both possible to describe situations where this interaction is high (*r*>>0, for example parent-offspring communication) or situations where there is no relatedness and the players do not interact with each other outside the signalling game (i.e. *r* = 0). Based on these assumptions the inclusive fitness of the signaller (*E*_*s*_) and the receiver (*E*_*r*_) can be written as follows:

$$E_{r}=W(a,z)-C_{r}(b_{r})+r\left(V(a,z)-C(a,b_{s})-C_{a}(a,b_{s})\right)$$ (3)

$$E_{s}=V(a,z)-C(a,b_{s})-C_{a}(a,b_{s})+r\left(W(a,z)-C_{r}(b_{r})\right)$$ (4)

**Fig 1:**
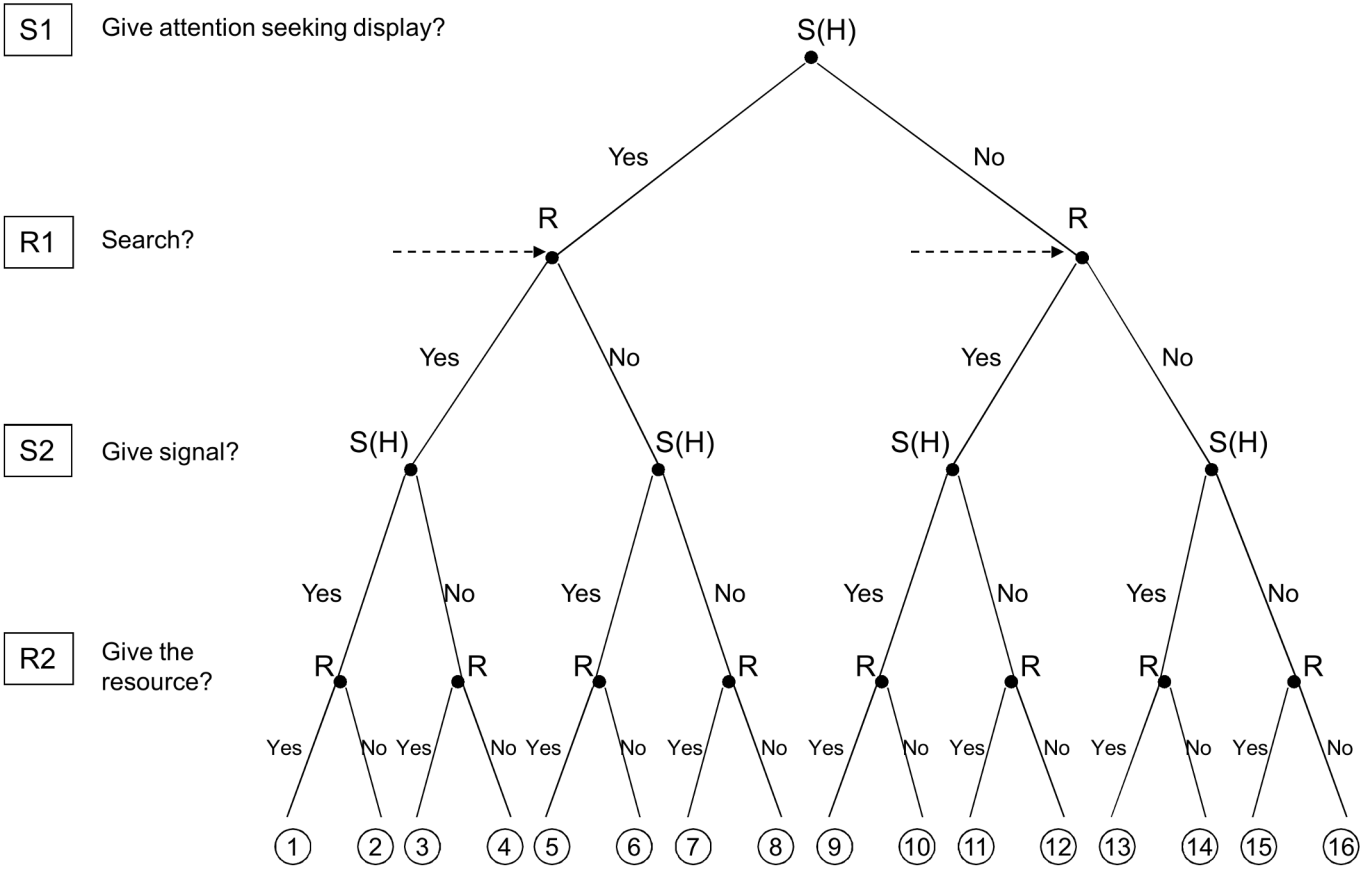
Extensive form of the signalling game. S1, S2 and R1, R2 denote the decisions the signaller and the receiver have to make respectively; S1: ASD stage, give or not to give an ASD; R1: search stage: search or not to search for ASDs; S2: quality signalling stage: give or not to give a quality signal; R2: decision stage: give or not to give the resource to the signaller. R and S(H) denote receiver and high quality signaller respectively. For the sake of simplicity the tree does not show the decision made by nature, i.e. the step that specifies the quality of signaller, it only shows the game for high quality signallers. The same decision tree can be drawn for low quality signallers. Dotted arrows denote the nodes where the receiver cannot tell whether the node is part of the first or the second tree, i.e. where the receiver cannot tell whether the signal (or lack of it) comes from a high or a from a low quality signaller. Table 2 gives the fitness values corresponding to the end nodes.

Note that *W*, *V* and *C* can each be defined as a function of the signaller’s state; i.e. *V*_*h*_ = *V*(*H*,*U*)-*V*(*H*,*D*), *V*_*l*_ = *V*(*L*,*U*)-*V*(*L*,*D*), where *V*_*h*_ denotes the difference in fitness for a high quality signaller between getting and not getting the resource (i.e. ‘up’ vs. ‘down’); and *V*_*l*_ denotes the same fitness difference for low quality signallers. The same differential can be calculated for the receiver’s fitness (*W*) and for the cost functions (*C*, *C*_*a*_ and *C*_*r*_) (see Table 1); where *W*_*h*_ denotes the fitness difference between giving and not giving the resource to a high quality signaller for a receiver, *W*_*l*_ denotes the same fitness difference in case of a low quality signaller; and finally *C*, *C*_*a*_ and *C*_*r*_ denote the cost difference between signalling/searching and not signalling/searching. This notation will be used in the rest of the article (see Table 1. for a summary). Let *x* denote the proportion of high quality signallers in the population. The signaller’s quality is originally hidden from the receiver but can be assessed upon investigating the signaller at close range. To do so first the signallers have to call the attention of the receivers. Accordingly, the game is a two-stage action- response game where the first stage is the search and the second stage is quality signalling (see Fig 1). During the first stage (S1) signallers can give a signal to call the attention of the receivers, this signal has a cost *C*_*a*_. Here we assume that the receiver has to make a decision (R1) between to search or not to search and that search has a cost (*C*_*r*_) and not searching will diminish the receiver’s chances to locate a signaller with *α* (0≤*α*<1). Let *p* denote the chance that the receiver locates the signaller in the presence of an ASD successfully, as opposed to the case when receivers have to locate the potential signallers in the absence of ASDs (*q*). It is worth to note that at this point receivers face an informational uncertainty as much as they do not know as yet the quality of the signaller, they found (see Fig 1). The receiver visits and assesses the signallers that were noticed. In the simplest case, which will be investigated here, the receiver visits only one signaller. Let us assume that once the signaller is found the receiver can assess the relevant quality reliably, i.e. this assessment (R2) is either based on indices or honest signals (S2); and conditions of the honest signalling equilibrium hold.

**Table 1:** Variables, parameters and notations of the model.

*F*_*r*_	receivers’ fitness
*F*_*s*_	signaller’s fitness
W	value of the receiver’s response for the receiver
*V*	value of the receiver’s response for the signaller
*C*	cost of signalling
*C*_*a*_	cost of the attention-seeking display
*C*_*r*_	cost of search
*a*	signaller’s quality
*b*_*s*_	signaller’s behaviour (signal vs. not to signal)
*b*_*as*_	signaller’s behaviour (give ASD vs. not to give)
*b*_*r*_	receiver’s behaviour (search vs. not to search)
*z*	receiver’s response (Up vs. down)
*r*	degree of interaction outside the signalling game
*p*	the probability that the receiver locates the signaller in the presence of an ASD
*q*	the probability that the receiver locates the signaller in the absence of an ASD
*α*	the probability that the receiver locates the signaller without searching for ASDs
*x*	the proportion of high quality signallers in the population
*H*	high quality signaller
*L*	low quality signaller
*U*	up, to give the resource
*D*	down, not to give the resource
*S*	signal
*N*	not to signal
*SA*	search for ASDs
*NS*	not to search for ASDs
*V*_*h*_ = *V*(*H*,*U*)-*V*(*H*,*D*)	difference in the value of the receiver’s responses for high quality signallers
*V*_*l*_ = *V*(*L*,*U*)-*V*(*L*,*D*)	difference in the value of the receiver’s responses for low quality signallers
*W*_*h*_ = *W*(*H*,*U*)-*W*(*H*,*D*)	difference in the value of the receiver’s responses for receivers in case of high quality signallers
*W*_*l*_ = *W*(*L*,*U*)-*W*(*L*,*D*)	difference in the value of the receiver’s responses for receivers in case of low quality signallers
*C*_*h*_ = *C*(*H*,*S*)-*C*(*H*,*N*)	difference in the cost of signals for high quality signallers
*C*_*l*_ = *C*(*L*,*S*)-*C*(*L*,*N*)	difference in the cost of signals for low quality signallers
*C*_*ah*_ = *C*_*a*_(*H*,*S*)-*C*_*a*_(*H*,*N*)	difference in the cost of attention-seeking displays for high quality signallers
*C*_*al*_ = *C*_*a*_(*L*,*S*)-*C*_*a*_(*L*,*N*)	difference in the cost of attention-seeking displays for low quality signallers
*Cr* = *C*_*r*_(*SA*)-*C*_*r*_(*NS*)	difference in the cost of search for receivers

The second stage describes this assessment (i.e. quality signalling), accordingly, the last step is to specify the pure strategies available to the players during this stage. The signaller can choose its strategy as a function of its state, whereas the receiver's strategy is a function of the signaller's behaviour. It follows that four pure strategies are available both to the signaller and to the receiver respectively. For the signaller each strategy pair describes what to do if High, what to do if Low: (i) always signal (S, S), (ii) signal only when High (S, N), (iii) signal only when Low (N, S) and (iv) never signal (N, N).; For the signaller each strategy pair describes what to do if the signaller signals, what to do if not: (i) always give the resource (U,U), (ii) only give the resource if the signaller signals (U,D), (iii) give the resource only if the signaller does not signal (D,U) and (iv) never give the resource (D,D). However, communication occurs in only four of the strategy pairs, when the signaller provides a variable signal and the receiver responds selectively to it. These four cases are formally identical, hence the same analysis can be carried out for each pair, thus, it is enough to analyse only one of them [(S,N) (U,D)] [5,6]. The game can be written up in extensive form as can be seen in Fig 1, Table 2 gives the pay-off values corresponding to the end nodes of the graph. These pay-off values follow the structure of Eqs 3 and 4 for the receiver and the signaller respectively. For example, at node 1 of Fig 1 high quality signallers give both ASD and quality signal and receivers do search for ASDs and give the resource; accordingly receivers find signallers with probability *p* and they transfer the resource (‘up’ response), and miss them with probability (1-*p*) and do not transfer it (see Table 1. 1^st^ row *L*_*r*_ and *L*_*s*_ respectively). As opposed to this, at node 5 while signallers give both kind of signals receivers do not search for ASDs; as a result the chance of finding signallers is decreased by *α*, thus, receivers find signallers and transfer the resource with probability *α p*, and miss them and do not transfer with probability (1- *αp*) (Table 1. 5^th^ row). In the last example, at node 10 signallers do not give ASDs but they give a quality signal, while receivers search for ASDs (thus pay the cost of it) yet they do not hand over the resource; accordingly the chance of finding a signaller (in the absence of ASD) is *q*, however, signallers get ‘down’ response (and fitness accordingly) regardless whether they are found or not (Table 1. 10^th^ row).

**Table 2:** The fitness values corresponding to the end nodes in Fig 1, where *Es* and *Er* denote the inclusive fitness of the signaller and the receiver respectively. The fitness of both players is a combination of the benefit they receive as a result of the receiver’s decision and the costs they have to pay depending on their own decisions (e.g. signal or not to signal, search or not to search, etc.)The same table can be drawn for low quality signallers (by substituting H with L).

End node (Fig 1.)	Receiver’s and Signaller’s fitness respectively
**1,**	*Er = P*(*W*(*H*,*U*)+*rV*(*H*,*U*))+(1*-p*)(*W*(*H*,*D*)+*rV*(*H*,*D*))-*C*_*r*_(*SA*)-*r*(*C*(*H*,*S*)+*C*_*a*_(*H*,*S*))
	*Es = p*(*V*(*H*,*U*)+*rW*(*H*,*U*))+(1*-p*)(*V*(*H*,*D*)+*rW*(*H*,*D*))-*C*(*H*,*S*)-*C*_*a*_(*H*,*S*)+*C*_*a*_(*H*,*S*)-*rC*_*r*_(*SA*)
**2,**	*Er = P*(*W*(*H*,*D*)+*rV*(*H*,*D*))+(1*-p*)(*W*(*H*,*D*)+*rV*(*H*,*D*))-*C*_*r*_(*SA*)-*r*(*C*(*H*,*S*)+*C*_*a*_(*H*,*S*))
	*Es = p*(*V*(*H*,*D*)+*rW*(*H*,*D*))+(1*-p*)(*V*(*H*,*D*)+*rW*(*H*,*D*))-*C*(*H*,*S*)-*C*_*a*_(*H*,*S*)-*rC*_*r*_(*SA*)
**3,**	*Er = P*(*W*(*H*,*U*)+*rV*(*H*,*U*))+(1*-p*)(*W*(*H*,*D*)+*rV*(*H*,*D*))-*C*_*r*_(*SA*)-*r*(*C*(*H*,*N*)+*C*_*a*_(*H*,*S*))
	*Es = p*(*V*(*H*,*U*)+*rW*(*H*,*U*))+(1*-p*)(*V*(*H*,*D*)+*rW*(*H*,*D*))-*C*(*H*,*N*)-*C*_*a*_(*H*,*S*)-*rC*_*r*_(*SA*)
**4,**	*Er = P*(*W*(*H*,*D*)+*rV*(*H*,*D*))+(1*-p*)(*W*(*H*,*D*)+*rV*(*H*,*D*))-*C*_*r*_(*SA*)-*r*(*C*(*H*,*N*)+*C*_*a*_(*H*,*S*))
	*Es = p*(*V*(*H*,*D*)+*rW*(*H*,*D*))+(1*-p*)(*V*(*H*,*D*)+*rW*(*H*,*D*))-*C*(*H*,*N*)-*C*_*a*_(*H*,*S*)-*rC*_*r*_(*SA*)
**5,**	*Er = αp*(*W*(*H*,*U*)+*rV*(*H*,*U*))+(1-*αp*)(*W*(*H*,*D*)+*rV*(*H*,*D*))-*C*_*r*_(*NS*)-*r*(*C*(*H*,*S*)+*C*_*a*_(*H*,*S*))
	*Es = αp*(*V*(*H*,*U*)+*rW*(*H*,*U*))+(1-*αp*)(*V*(*H*,*D*)+*rW*(*H*,*D*))-*C*(*H*,*S*)+*C*_*a*_(*H*,*S*)-*rC*_*r*_(*NS*)
**6,**	*Er = αp*(*W*(*H*,*U*)+*rV*(*H*,*D*))+(1-*αp*)(*W*(*H*,*D*)+*rV*(*H*,*D*))-*C*_*r*_(*N*,*S*)-*r*(*C*(*H*,*S*)+*C*_*α*_(*H*,*S*))
	*Es = αp*(*V*(*H*,*U*)+*rW*(*H*,*D*))+(1-*αp*)(*V*(*H*,*D*)+*rW*(*H*,*D*))-*C*(*H*,*S*)-*C*_*a*_(*H*,*S*)-*rC*_*r*_(*NS*)
**7,**	*Er = αp*(*W*(*H*,*U*)+*rV*(*H*,*D*))+(1-*αp*)(*W*(*H*,*D*)+*rV*(*H*,*D*))-*C*_*r*_(*N*,*S*)-*r*(*C*(*H*,*N*)+*C*_*α*_(*H*,*S*))
	*Es = αp*(*V*(*H*,*U*)+*rW*(*H*,*U*))+(1-*αp*)(*V*(*H*,*D*)+*rW*(*H*,*D*))-*C*(*H*,*N*)-*C*_*a*_(*H*,*S*)-*rC*_*r*_(*NS*)
**8,**	*Er = αp*(*W*(*H*,*D*)+*rV*(*H*,*D*))+(1-*αp*)(*W*(*H*,*D*)+*rV*(*H*,*D*))-*C*_*r*_(*NS*)-*r*(*C*(*H*,*N*)+*C*_*α*_(*H*,*S*))
	*Es = αp*(*V*(*H*,*D*)+*rW*(*H*,*D*))+(1-*αp*)(*V*(*H*,*D*)+*rW*(*H*,*D*))-*C*(*H*,*N*)-*C*_*a*_(*H*,*S*)-*rC*_*r*_(*NS*)
**9,**	*Er = q*(*W*(*H*,*U*)+*rV*(*H*,*U*))+(1-*q*)(*W*(*H*,*D*)+*rV*(*H*,*D*))-*C*_*r*_(*SA*)-*r*(*C*(*H*,*S*)+*C*_*α*_(*H*,*N*))
	*Es = q*(*V*(*H*,*U*)+*rW*(*H*,*U*))+(1-*q*)(*V*(*H*,*D*)+*rW*(*H*,*D*))-*C*(*H*,*S*)-*C*_*α*_(*H*,*N*)-*rC*_*r*_(*SA*)
**10,**	*Er = q*(*W*(*H*,*D*)+*rV*(*H*,*D*))+(1-*q*)(*W*(*H*,*D*)+*rV*(*H*,*D*))-*C*_*r*_(*SA*)-*r*(*C*(*H*,*S*)+*C*_*α*_(*H*,*N*))
	*Es = q*(*V*(*H*,*D*)+*rW*(*H*,*D*))+(1-*q*)(*V*(*H*,*D*)+*rW*(*H*,*D*))-*C*(*H*,*S*)-*C*_*α*_(*H*,*N*)-*rC*_*r*_(*SA*)
**11,**	*Er = q*(*W*(*H*,*U*)+*rV*(*H*,*U*))+(1-*q*)(*W*(*H*,*D*)+*rV*(*H*,*D*))-*C*_*r*_(*SA*)-*r*(*C*(*H*,*N*)+*C*_*α*_(*H*,*N*)
	*Es = q*(*V*(*H*,*U*)+*rW*(*H*,*U*))+(1-*q*)(*V*(*H*,*D*)+*rW*(*H*,*D*))-*C*(*H*,*N*)-*C*_*α*_(*H*,*N*)-*rC*_*r*_(*SA*)
**12,**	*Er = q*(*W*(*H*,*D*)+*rV*(*H*,*D*))+(1-*q*)(*W*(*H*,*D*)+*rV*(*H*,*D*))-*C*_*r*_(*SA*)-*r*(*C*(*H*,*N*)+*C*_*α*_(*H*,*N*))
	*Es = q*(*V*(*H*,*D*)+*rW*(*H*,*D*))+(1-*q*)(*V*(*H*,*D*)+*rW*(*H*,*D*))-*C*(*H*,*N*)-*C*_*α*_(*H*,*N*)-*rC*_*r*_(*SA*)
**13,**	*Er = αq*(*W*(*H*,*U*)+*rV*(*H*,*U*))+(1-*αp*)(*W*(*H*,*D*)+*rV*(*H*,*D*))-*C*_*r*_(*NS*)-*r*(*C*(*H*,*S*)-*C*_*a*_(*H*,*N*)
	*Es = αq*(*V*(*H*,*U*)+*rW*(*H*,*U*))+(1-*αq*)(*V*(*H*,*D*)+*rW*(*H*,*D*))-*C*(*H*,*S*)-*C*_*a*_(*H*,*N*)-*rC*_*r*_(*NS*)
**14,**	*Er = αq*(*W*(*H*,*D*)+*rV*(*H*,*D*))+(1-*αp*)(*W*(*H*,*D*)+*rV*(*H*,*D*))-*C*_*r*_(*NS*)-*r*(*C*(*H*,*S*)-*C*_*a*_(*H*,*N*)
	*Es = αq*(*V*(*H*,*D*)+*rW*(*H*,*D*))+(1-*αq*)(*V*(*H*,*D*)+*rW*(*H*,*D*))-*C*(*H*,*N*)-*C*_*α*_(*H*,*N*)-*rC*_*r*_(*NS*)
**15,**	*Er = αq*(*W*(*H*,*U*)+*rV*(*H*,*U*))+(1-*αq*)(*W*(*H*,*D*)+*rV*(*H*,*D*))-*C*_*r*_(*NS*)-*r*(*C*(*H*,*N*)+*C*_*a*_(*H*,*N*))
	*Es = αq*(*V*(*H*,*U*)+*rW*(*H*,*U*))+(1-*αq*)(*V*(*H*,*D*)+*rW*(*H*,*D*))-*C*(*H*,*N*)-*C*_*α*_(*H*,*N*)-*rC*_*r*_(*NS*)
**16,**	*Er = αq*(*W*(*H*,*D*)+*rV*(*H*,*D*))+(1-*αq*)(*W*(*H*,*D*)+*rV*(*H*,*D*))-*C*_*r*_(*NS*)-*r*(*C*(*H*,*N*)+*C*_*a*_(*H*,*N*))
	*Es = αq*(*V*(*H*,*D*)+*rW*(*H*,*D*))+(1-*αq*)(*V*(*H*,*D*)+*rW*(*H*,*D*))-*C*(*H*,*N*)-*C*_*α*_(*H*,*N*)-*rC*_*r*_(*NS*)

## Results

We are searching for an ESS pair of signaller and receiver strategies, where the quality signalling stage is honest i.e. signallers signal only when High and receivers only give the resource if the signaller signals [(S,N) (U,D)] and at least high quality signaller give ASDs and receivers search for it. The ESS condition implies that neither the receiver nor the signaller wishes to deviate from the corresponding strategy assuming that this strategy pair is played in the population [21]. This means that the use of ASD and the use of search is a Nash equilibrium (NE) for signallers and receivers respectively. More precisely we are looking for a sub-game perfect NE (SPNE) that specifies actions for each sub-game of the tree. Basically, this is a stability criterion for extensive form games, as a non sub-game perfect NE is not an ESS since it can be invaded by neutral mutants [22]. A sub-game perfect NE can be found by using backwards induction [22,23,24]. One should start at the minimal sub-game and work his/her way upward on the tree searching for the NE of each truncated game. In our case this means that the path leading from node 1 where high quality signallers use ASD and receivers search and the path leading from node 12 where low quality signallers do not use ASD and receivers search should be NE of each truncated game as we move upwards for high and for low quality signaller respectively. This means that the SPNE for receivers and signallers can be described as follows: [((S,N) (SA)), ((S,N) (U,D))], where the first main bracket specifies the signaller and receiver strategies at the first attention-seeking stage (i.e. R1/S1 nodes of Fig 1) and the second main bracket specifies the signaller and receiver strategies at the quality signalling stage (R2/S2 nodes of Fig 1). The first main bracket implies that signallers only give attention-seeking displays if they are High and receivers search for it (it is a simple bracket because receivers at this stage have no cue or signal to base their decisions on); and the second main bracket implies that signallers signal only when High and receivers only give the resource if the signaller signals.

**Fig 2:**
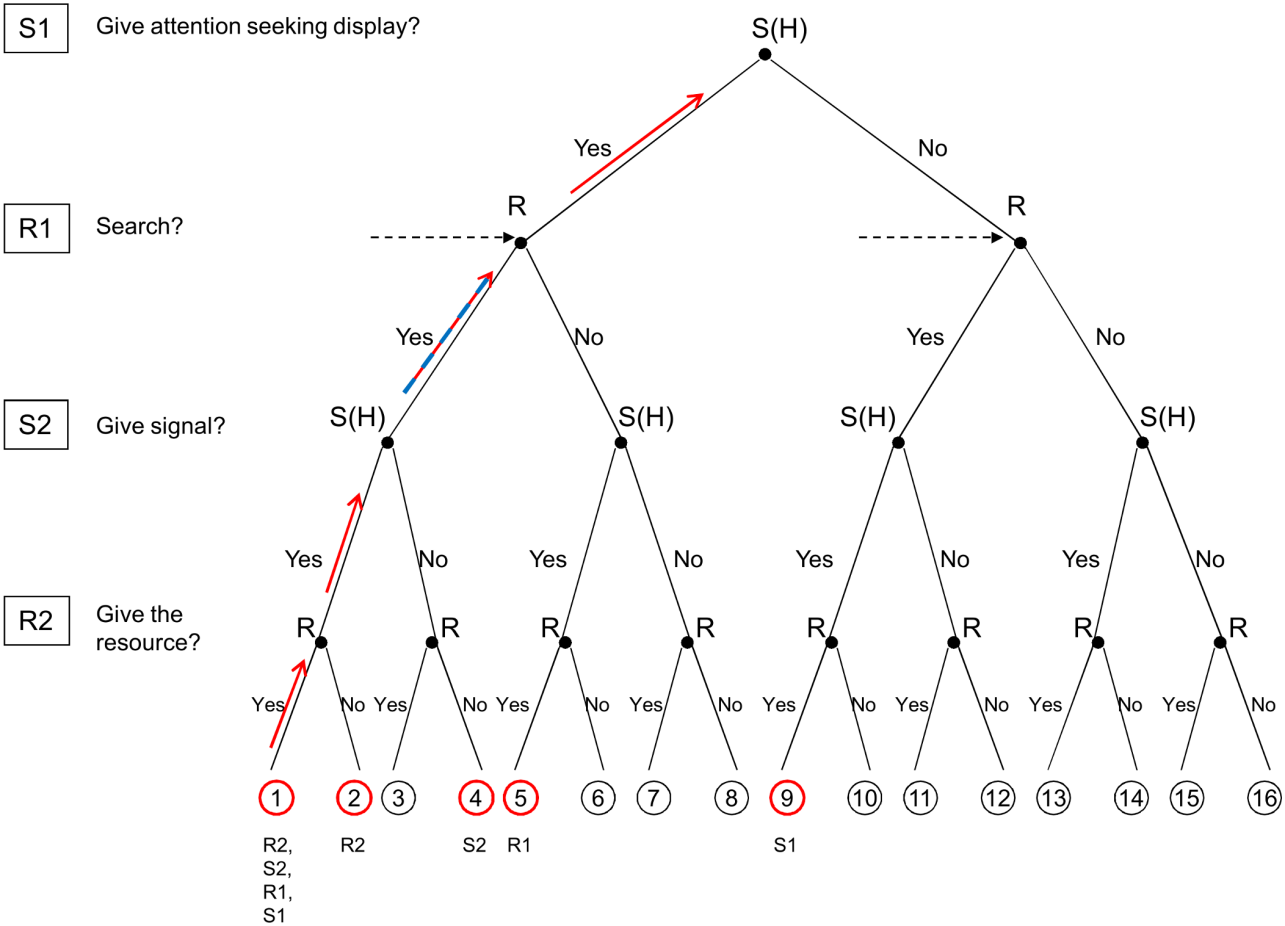
The logic of backward induction for high quality signallers. Arrows show the progression, coloured circles show the compared nodes and the label(s) below the circles show the step at which the given nodes are compared. We are searching for a pair of ESS strategies that lead to node 1 in case of the high quality signaller (i.e. where receivers search and high quality signallers give ASD). First step: node 1 vs. node 2 from the receiver’s point of view (R2). Second step: node 1 vs. node 4 from the signaller’s point of view (S2; note, since we are interested in the stability of the receiver's strategy of playing (U,D), strategy node 3 does not need to be considered). Third step: nodes 1 and 5 vs. nodes 12 and 16 weighted by the ratio of high to low quality individuals from the receiver’s point of view (R1; the reason behind comparing several nodes is the informational uncertainty the receiver facing at this point). Fourth step: node 1 vs. node 9 from the signaller’s point of view (S1).

**Fig 3:**
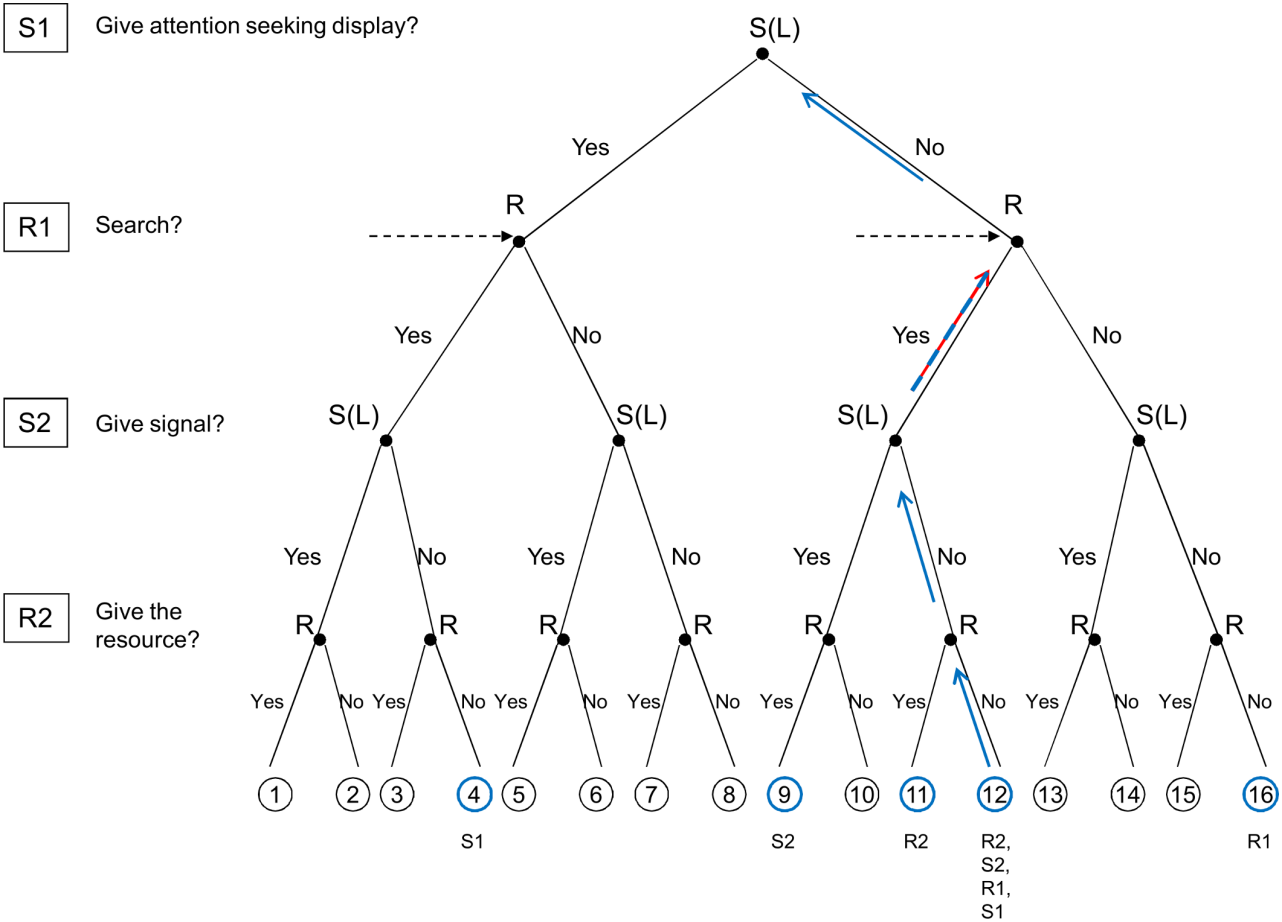
The logic of backward induction for low quality signallers. Arrows show the progression, coloured circles show the compared nodes and the label(s) below the circles show the step at which the given nodes are compared. We are looking for a pair of ESS strategies that lead to node 12 in case of low quality signallers (i.e. where receivers search but they do not give the resource to low quality signallers, and accordingly, low quality signallers do not give signals of quality). First step: node 11 vs. node 12 from the receiver’s point of view (R2). Second step: node 9 vs. node 12 from the signaller’s point of view (S2; note, since receivers have an (U,D) strategy node 10 is not obtainable). Third step: nodes 1 and 5 vs. nodes 12 and 16 weighted by the ratio of high to low quality individuals from the receiver’s point of view (R1; the reason behind comparing several nodes is the informational uncertainty the receiver facing at this point). Fourth step: node 4 vs. node 12 from the signaller’s point of view (S1).

In the minimal sub-game (at the level of R2, node 1 vs. node 2 and node 11 vs. node 12 respectively) it is the receiver’s criteria [6] that should hold (see Figs 2 and 3 for high and low quality signallers respectively). These criteria simply states the receivers are better off giving the resource to high quality signallers than not; on the other hand, they are better off withholding the resource from low quality ones. Formally, it is as follows (see Appendix 1 for details on this calculation and for all the further calculations):

$$W_{h}+rV_{h}>0,$$ (5) (A.1)

$$W_{l}+rV_{l}<0.$$ (6) (A.2)

In other words, the fitness difference between giving and not giving the resource to high quality signallers should be positive (i.e. it is worth giving, Eq 5), while the same fitness difference for low quality signallers is negative (Eq 6). In the next sub-game (S2, node 1 vs. node 4 and node 9 vs. node 12 respectively) it is the signaller’s criteria that should hold [6]. Note, we compare nodes 1 vs. 4 and not 1 vs. 3, because we are interested in honest signalling: under this assumption the receiver will not transfer the resource when the signaller gives no signal (i.e. receivers have an (U,D) strategy), thus node 3 is not obtainable. The same logic holds for node 9 as well: since receivers have an (U,D) strategy node 10 is not obtainable. This tells that high quality signallers are better off giving a signal, while low quality ones prefer not to signal (i.e. the signaller strategy at this stage should be (S,N)). It can be written as follows:

$$p\left(V_{h}+rW_{h}\right)>C_{h},$$ (7) (A.3)

$$q\left(V_{l}+rW_{l}\right)<C_{l}.$$ (8) (A.4)

This implies that the benefits of getting the resource weighted by the chance that the receiver finds the signaller should be larger than the cost of signalling (i.e. the cost difference between giving and not giving a signal) for high quality signallers (Eq 7), and the same benefits should be lower than the cost of signalling for low quality signallers (Eq 8). Both the result of this level and the previous one (Eqs 5–8) follow from the fact that we assume honest signalling of quality; accordingly, assuming *p* = 1 and *q* = 1 (i.e. that there is no searching stage) we got back the result of standard action-response games (in other words the receiver’s and the signaller’s criteria specifies a sub-game perfect NE in action response games, [4,5,6]).

The next level is the search stage for receivers (R1). The choice to search in the truncated game (assuming honest signalling) has to be a NE. The situation is a bit more complicated here than in the previous two cases because the receiver does not know which type of signaller will be found as a result of the search (i.e. there is an informational uncertainty, see Fig 1 R1 decision nodes, noted by dotted arrows). Accordingly, the condition has to be checked on both trees (node 1 vs. node 5 and node 12 vs. node 16 respectively) and weighted by the frequency of each type. The choice to search is a NE if the following condition is satisfied:

$$xp\left(1-\alpha \right)(W_{h}+rV_{h})>C_{r}$$ (9) (A.5)

That is, the benefit that results from finding a high quality signaller have to be larger than the cost of searching. Note that these benefits are the highest when *α* = 0, i.e. when receivers cannot find signallers (at all) without searching for ASDs. On the other hand, when receivers can perfectly locate signallers without searching for ASDs (i.e. *α* = 1) benefits from search disappear, thus the above condition cannot be satisfied. The last level is the attention-seeking display stage for the signallers (S1). Then, for a high quality male the following condition has to hold in order for signalling with ASD to be NE (node 1 vs. node 9):

$$(p-q)(V_{h}+rW_{h})>C_{ah}.$$ (10) (A.6)

This means that the potential benefits of signalling weighted by the difference the ASD makes in finding a mate has to be larger than the cost of an ASD. For low quality males we assume that not giving ASD is the NE (i.e. node 12, Fig 1), then the following condition must hold (assuming that receivers search at R1 but they do not give the resource to low quality signallers at R2, i.e. node 4 vs. node 12):

$$0<C_{al}.$$ (11) (A.7)

We can see that as long as ASD has a cost this condition will be always satisfied. That is, the combination of giving ASD for high quality signallers, not giving them for low quality ones and finally searching for receivers will be the ESS (i.e. SPNE) as long as Eq 9, Eq 10 and Eq 11 are satisfied.

**Fig 4:**
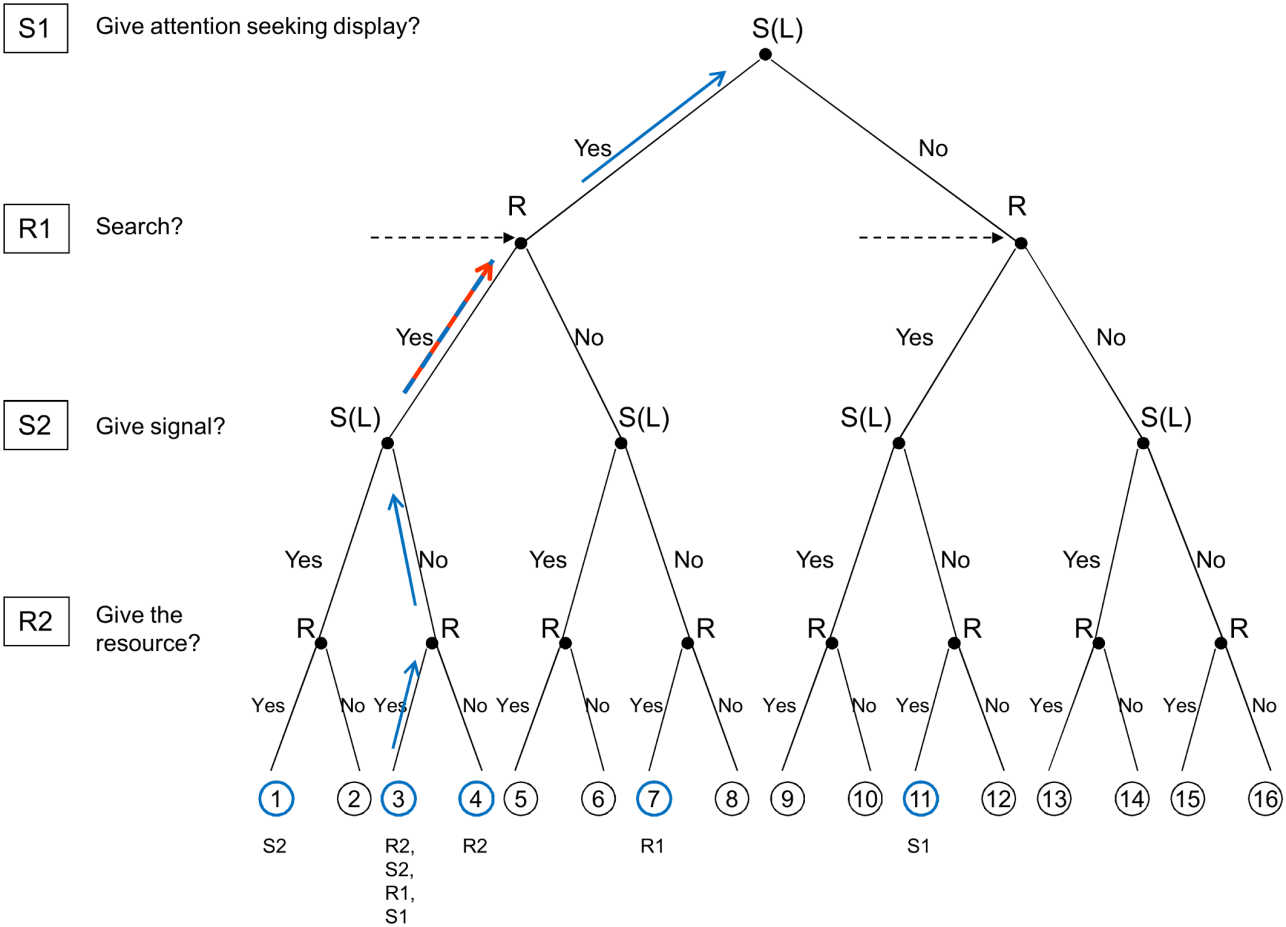
The logic of backward induction for low quality signallers when it is worth for the receiver to give the resource to low quality signallers in the absence of high quality ones. Arrows show the progression, coloured circles show the compared nodes and the label(s) below the circles show the step at which the given nodes are compared. We are looking for a pair of ESS strategies that lead to node 3 in case of low quality signallers (i.e. where receivers search and they give the resource to low quality signallers in the absence of high quality ones, however low quality signallers still do not give signals of quality). First step: node 3 vs. node 4 from the receiver’s point of view (R2). Second step: node 1 vs. node 3 from the signaller’s point of view (S2). Third step: nodes 1 and 5 vs. nodes 3 and 7 weighted by the ratio of high to low quality individuals from the receiver’s point of view (R1; the reason behind comparing several nodes is the informational uncertainty the receiver facing at this point). Fourth step: node 3 vs. node 11 from the signaller’s point of view (S1).

Note that in the original action-response game [5] it is assumed that it is not worth giving the resource to low quality signallers for receivers in the absolute sense; i.e. they receive a negative pay-off if they do so. This might not be the case in nature. Low quality signallers might give some benefit to the receiver-i.e. it might still be worth mating with a low quality mate in the absence of high quality ones rather than to forfeit mating. Formally it means that node 3 must be a sub-game perfect NE, in other words it should be NE of each truncated game as we move upwards for low quality signallers (Fig 4). Thus, the SPNE for receivers and signallers is modified follows: receiver|S(H) (search, up), receiver|S(L) (search, up), signaller|S(H) (signal, signal), and signaller|S(L) (signal, not to signal), Then the receiver’s criteria (R2) is as follows (node 3 vs. 4):

$$W_{l}+rV_{l}>0.$$ (12) (A.8)

This means that the receiver’s fitness is above zero when transferring the resource to a low quality signaller (vs. Eq 6). However, the receiver is still better off giving the resource to a high quality one, thus:

$$W_{h}+rV_{h}>W_{l}+rV_{l}.$$ (13)

That is, the fitness difference between giving and not giving the resource to high quality signallers is higher than the same difference for low quality signallers. The signaller’s criteria (S2) is unchanged since we still assume that the quality signalling stage is honest, i.e. low quality individuals do not give a signal.

At the search stage (R1) we assume that the SPNE is to search, thus receivers should be better off searching than not, accordingly, this will result the following changes (node 1 vs. node 5 and node 3 vs. node 7 weighted by the frequency of high and low quality signallers):

$$p\left(1-\alpha \right)\left[x(W_{h}+rV_{h})+(1-x)(W_{l}+rV_{l})\right]>C_{r}$$ (14) (A.9)

That is, the benefit that results from finding a signaller have to be larger than the cost of searching, where the benefits resulting from the transfer of the resource to high and low quality signallers are weighted by their proportion in the population (*x* and 1-*x* respectively). Note that just as in case of Eq 9. the probability of finding signallers with ASDs (*p*) is weighted by 1-*α*, so the same conclusions apply as before. At the stage of ASD (S1) we assume that it is worth to give ASD for low quality signallers too and this results the following condition (node 3 vs. node 11):

$$(p-q)(V_{l}+rW_{l})>C_{al},$$ (15) (A.10)

We can see that the potential benefits of signalling weighted by the difference that giving the ASD means have to be larger than the cost of ASD for low quality signallers.

This means that if all of the above conditions (Eqs 12–15) are satisfied alongside Eq 10, then both low and high quality signallers will give ASD, and search is the ESS for receivers. Moreover, ASD will remain honest in the sense that it advertises the presence (availability) of potential partners (for mating or for other purposes) and not the quality of them.

In order to facilitate the interpretation of the model I give four different parametrizations using examples from the literature. The first one is the classic Sir Philiph Sidney game (JMS91) [4], the second one used by Bergstrom and Lachmann (BL97) [25] and the last one used by Polnaszek and Stephens (PS14) [26] in their experimental model testing the role of signal cost in honesty. These examples differ in their parametrizations and in terms of notations and pay-offs. Table 3 gives the notation of these models and how they relate to the terminology of the current model. The first two examples [4,25] are resource sharing models where the donor receives no direct benefit from giving the resource to the signaller; accordingly the donor’s fitness is reduced in both case (1-*S* and *d* respectively, where 0<*S*,*d*<1; [4,25]). However, the donors gain indirectly in both model [4,25] by being linked (‘related’) to the signaller’s fitness outside of the signalling interaction. As noted in the introduction this need not be biological relatedness, though it is the most obvious example [4]. It could simply mean that they belong to the same group; hence their survival will influence the survival of the others in a positive way. On the other hand, the last model [26] assumes that the donor receives some direct benefit as a result of the resource transfer: an assumption most likely to apply to mate choice. As a result, donors get the highest pay-off when either they transfer the resource to high quality signallers, or do not transfer it to low quality ones ([26], see Table 3). It also means that positive *r* is not necessary in this case; and indeed *r* = 0 is assumed by PS14 [26]. Signallers in all three examples benefit from the transfer of the resource, just high and low quality ones benefit differentially (i.e. all three examples are differential benefit models). Low quality signallers benefit less in all three examples, where the difference is *V*, *a*-*b*, 1-*a*+*b* respectively [4,25, 26]. The signal cost is the same for low and high quality signallers in all three models (*t*,*c*,*c* respectively). Since there are no ASDs and no search for them, I use the current notation of these functions. Last but not least, I give a differential cost example. In this case the benefits are the same for high and low quality signallers (1); it is the signal cost that differs: *c* and *d* for low and high quality signallers respectively, where *c*>*d*. Let’s assume that donors receive some direct benefit transferring the resource (like PS14 [26]), thus they get the highest benefit when they transfer the resource to high quality signallers and do not transfer to low quality ones (Table 3). Lastly, the assumption *r* = 0 used just like in PS14 [26]. The SPNE conditions for all these examples are summarized in Table 4. The second type of equilibrium (when low quality signallers also give ASDs) is not possible in the last two cases because Eq 12 cannot be fulfilled. It is clear that it is possible to create very different parametrizations, some of them can allow both type of equilibria studied in the model, some of them cannot.

**Table 3:** Parametrization of the general model using three different action-response games [4,25, 26].

General notation	JMS91	BL97	PS14	Differential cost model
V(H,U)	1	1	1	1
V(H,D)	0	1-*a*	0	0
V(L,U)	1	1	*a*	1
V(L,D)	*V*	1-*b*	*b*	0
W(H,U)	*S*	1-*d*	1	1
W(H,D)	1	1	0	0
W(L,U)	*S*	1-*d*	0	0
W(L,D)	1	1	1	1
C(H,S)	*t*	*c*	*c*	*d*
C(H,N)	0	0	0	0
C(L,S)	*t*	*c*	*c*	*c*
C(L,N)	0	0	0	0
*V*_*h*_ = *V*(*H*,*U*)-*V*(*H*,*D*)	1	a	1	1
*V*_*l*_ = *V*(*L*,*U*)-*V*(*L*,*D*)	1-*V*	b	*a*-*b*	1
*W*_*h*_ = *W*(*H*,*U*)-*W*(*H*,*D*)	*S*-1	-*d*	1	1
*W*_*l*_ = *W*(*L*,*U*)-*W*(*L*,*D*)	*S*-1	-*d*	-1	-1
*C*_*h*_ = *C*(*H*,*S*)-*C*(*H*,*N*)	*t*	*c*	*c*	*d*
*C*_*l*_ = *C*(*L*,*S*)-*C*(*L*,*N*)	*t*	*c*	*c*	*c*

**Table 4:** SPNE conditions using the parametrization of three different action-response games [4,25, 26].

Eq	SPNE conditions	JMS91	BL97	PS14	Differential cost model
	1. Equilibrium				
5.	*W*_*h*_ *+rV*_*h*_>0	*S*-1+*r*>0	-*d*+*ra*>0	1+*r*>0	1+*r*>0
6.	*W*_*l*_+*rV*_*l*_<0	*S*-1+*r*(1-*V*)<0	-*d*+*rb*<0	-1+*r*(*a*-b)<0	-1+*r*>0
7.	*p*(*V*_*h*_+*rW*_*h*_)>*c*_*h*_	*p*(1+*r*(*S*-1))>*t*	*p*(*a*-*rd*)>c	*p*(1+*r*)>c	*p*(1+*r*)>*d*
8.	*q*(*V*_*l*_+*rW*_*l*_)<*C*_*l*_	*q*(1-*V*+*r*(*S*-1))<t	*q*(*b-rd*)<*c*	*q*(*a*-*b*-*r*)<*c*	*q*(1-*r*)<*c*
9.	*xp*(1-*α*)(*W*_*h*_ *+rV*_*h*_)>*C*_*r*_	*xp*(1-*α*)(*S-*1+*r*)>*C*_*r*_	*xp*(1-*α*)(*-d*+*ra*)>*C*_*r*_	*xp*(1-*α*)(1+*r*)>*C*_*r*_	*xp*(1-*α*)(1+*r*)>*C*_*r*_
10.	(*p*-*q*)(*V*_*h*_+*rW*_*h*_)>*C*_*ah*_.	(*p*-*q*)(1+*r*(*S*-1))>*C*_*ah*_	(*p*-*q*)(*a-rd*)>*C*_*ah*_	(*p-q*)(1+*r*)>*C*_*ah*_	(*p-q*)(1+*r*)>*C*_*ah*_
	SPNE conditions				
	2. Equilibrium				
12.	*W*_*l*_ *+rV*_*l*_>0	*S*-1+*r*(1-*V*)>0	-*d*+*rb*>0	It is not possible.	It is not possible.
13.	*W*_*h*_ *+rV*_*h*_>*W*_*l*_ *+rV*_*l*_	*S*-1+*r*>*S*-1+*r*(1-*V*)	-*d*+*ra*>-*d*+*rb*	NA	NA
14.	$\begin{array}{l} p\left(1-\alpha \right)\\ \left[\begin{array}{l} x(W_{h}+rV_{h})+\\ \left(1-x)(W_{l}+rV_{l}\right) \end{array}\right]>C_{r} \end{array}$	$\begin{array}{l} p\left(1-\alpha \right)\\ \left[\begin{array}{l} x(S-1+r)+\\ \left(1-x\right)\left(S-1r\left(1-V\right)\right) \end{array}\right]>C_{r} \end{array}$	$\begin{array}{l} p\left(1-\alpha \right)\\ \left[\begin{array}{l} x\left(-d+ra\right)+\\ \left(1-x\right)\left(-d+rb\right) \end{array}\right]>C_{r} \end{array}$	NA	NA
15.	(*p*-*q*)(*V*_*l*_+*rW*_*l*_)>*C*_*al*_	(*p*-*q*)(1-*V*+*r*(*S*-1))>*C*_*al*_	(*p*-*q*)(*b-rd*)>*C*_*al*_	NA	NA

## Discussion

Here I have shown that for high quality signallers giving attention-seeking displays and for receivers searching for them can be an ESS in an extended action-response game. The conditions of this equilibrium are very straightforward: (i) for receivers the benefit that results from finding a high quality signaller has to be larger than the cost of searching (Eq 9), these benefits are the highest when *α* = 0, i.e. when receivers cannot locate signallers without searching for ASDs. (ii) For high quality signallers the potential benefits of signalling resulting from the increased chance of detection due to the ASD has to be larger than the cost of giving an ASD (Eq 10). These benefits are the highest if *q* = 0, i.e. when receivers cannot find those signallers who do not give ASDs; in general *p*>>*q* favours the evolution of ASDs. It is also clear from Eqs 9 and 10 that high *r*, which means aligned interest, promotes the evolution of ASDs as it increases the potential benefits both for signallers and receivers. The existence of two possible signalling equilibria was shown for low quality signallers. In the first it is not worth for receivers to transfer the resource to low quality individuals, hence it is not worth for low quality signallers to give ASDs at all (Eq 11). Thus, in this first case the ASD reveals the quality of the signaller as well (since only high quality signallers give it at the equilibrium). However, this need not be always the case. In the second type of equilibrium-in the absence of high quality signallers- it is worth for the receiver to transfer the resource to low quality ones, thus it becomes beneficial to give ASD for the low quality signallers as well. The conditions of this second type of equilibrium are pretty straightforward too: (i) for receivers the benefit that results from finding a high or a low quality signaller have to be larger than the cost of searching (Eq 14), (ii) for high quality signallers the condition is the same as before (i.e. Eq 10), (iii) and finally, for low quality signallers the potential benefits of signalling resulting from the increased chance of detection due to the ASD has to be larger than the cost of giving an ASD (Eq 15). All in all, this means that ASDs need not be informative at the equilibrium regarding the quality of the signaller. Finally, these signals need not be costly at the equilibrium (as none of the conditions impose a lower limit on the observed equilibrium ASDs, see Eqs 10 and 15) but they could be very expensive as well (assuming that the benefits still outweight these costs), as the cost depends on the physical nature of the environment in which the competition takes place, i.e. it depends on those conditions under which attention must be secured.

This means in turn, that if the receiver is interested in any quality of signaller, and the ASD is non-revealing in this respect, then some other means have to be found to gather such information. That is, the receiver has to find potential indices, performance displays, costly signals, etc. related to the quality under question. This implies that under such situations multicomponent displays are expected to evolve [see 13]. ASDs signal the intention to compete and help the receiver to locate the signaller, while the other components are used to assess the quality of the signaller. This division of labour between display components has been previously suggested [13] and it is supported by empirical studies [16,17,18,19,20], and it offers an alternative explanation why we can find multi-component displays in nature compared to other explanations such as back-up signals or multiple-messages [27,28,29] or proposed non-equilibrium solutions [30].

Note that the theory of ASDs provides an alternative to the handicap principle [31] in explaining so called extravagant displays, and it also offers a different set of predictions. Namely, it predicts that: (1) ASDs may or may not be costly; (2) they may or may not reveal some relevant quality of the signaller; (3) when they do not reveal the relevant quality of the signaller then they should be part of a multicomponent display with signals of quality and or intent; (4) they should evolve to be highly detectable in the natural environment of the given species (i.e. *p*>>*q* promotes the evolution of ASDs–see Eq 10-, where *p* is related to detectability). This latest prediction is important because the handicap principle does not give any direct predictions about signal detectability. On the one hand, signal reliability (i.e. honesty) might be more important than detectability for signals of quality; on the other hand, signal detectability is more important than honesty for ASDs that do not reveal quality. All in all, following Richards [13] one can set up three criteria: (i) ASDs must be highly detectable; (ii) ASDs should be resistant to degradation during transmission; and finally (iii) ASDs should show high contrast to extraneous perturbations. In other words, the theory of attention-seeking displays puts signal detectability [32] into a strategic context and gives predictions about when and why signal detectability should be a major concern.

Many of the signals that were previously interpreted as costly signals of quality could be ASDs i.e. signals of intention to compete, instead of being honest signals of any quality. There is plenty of empirical evidence in support of this view as discussed in the Introduction. Alert displays are described for a number of species [14,15]; see [33] for a review. Studies also show that initial attraction and final mate choice can be influenced by different parts of multicomponent displays [16,17,18,19,20]. Last but not least, the theory of ASDs could help the interpretation of those studies that show that signals used in mate choice do not necessarily reveal any relevant quality of the signaller. For example, it was found that long-distance acoustic mate attraction calls do not correlate with courtship effort in Jamaican crickets, *Gryllus assimilis* [34], with male fertility and female preference [35], and with aggressiveness in spring field crickets, *Gryllus veletis* [36]; but see [34]. These later results received little attention as they were considered as negative evidence, and it is very difficult to gain full confidence that no relevant information is transmitted at all. However, the current theory might help to put these studies in a different light. These signals may function as ASDs which could explain the lack of information. These signals are all highly conspicuous–like the male’s red breeding colouration in three-spiked stickleback (*Gastorosteus acuelatus*) [37] or the acoustic signals of field crickets (*Gryllus campestris*) [38] or that of the drumming spider (*Hygrolycosa rubrofasciata*) [39]—and they all relate to the environment and to the sensory capabilities of the species in a way that makes them highly detectable. Another potential example of attention-seeking displays is the courtship of sand gobies (*Pomatoschistus minutus*). Males court very intensely, yet courtship intensity correlates neither with quality nor with mating success, as females do not prefer males with high intensity courtship displays [40]. Lehtonen explicitely argues that “attention gathering may be especially important for a species such as the sand gobi, in which males are guarding inconspicuous, camouflaged nests” ([40], pp.1159).

Begging signals could provide another set of example for ASDs. Almost all of the begging signals are part of some multicomponent display and several empirical studies show that the function of these signals might differ. For example, the change of mouth colour (“red flush”) is a signal of need in the seed-regurgitating finches [41] while the pale colour of fringes increase the contrast of the mouth thus makes it more detectable for parents [41,42]. Moreover, this contrast is more important in dimly lit conditions [41,43]. Other examples cannot be excluded, all situations where signallers have to call the attention of receivers offer good grounds to expect the presence of attention-seeking displays.

Note that even though there is a potential conflict of interest, as it is not in the interest of the receivers to find low quality signallers, in the traditional sense it is not possible to cheat with ASDs. First of all, ASDs need not transmit information on the quality of the signaller. Second, the type of information transmitted by ASDs-i.e. location, intention to compete- cannot be dishonestly given in a meaningful way, at least not by the members of the same species. ASDs can be exploited though by third party members such parasites or predators, like the predatory fireflies [44].

All in all, giving and searching for attention-seeking displays can be an evolutionarily stable strategy in extensive form games. These signals may or may not reveal the quality of the signaller, may or may not be costly; however, they are expected to be highly detectable in the natural environment of the given species. Attention-seeking displays might be more prevalent in nature than their current weight in the literature suggests. Extravagant traits previously interpreted as costly signals of quality might turn out to be ASDs. Highly detectable signals given even in the absence of receivers, or given at the “introductory stage” of the interaction that transmit low or no information on the quality of the signaller are prime candidates for this function.

## Appendix 1

### Calculations of the ESS conditions

R2: The receiver’s criteria, high quality signallers (node 1 vs. 2):

$$\begin{array}{l} p\left(W\left(H,U\right)+rV\left(H,U\right)\right)+\left(1-p\right)\left(W\left(H,D\right)+rV\left(H,D\right)\right)-C_{r}\left(SA\right)-r\left(C\left(H,S\right)+C_{a}\left(H,S\right)\right)>\\ p\left(W\left(H,D\right)+rV\left(H,D\right)\right)+\left(1-p\right)\left(W\left(H,D\right)+rV\left(H,D\right)\right)-C_{r}\left(SA\right)-r\left(C\left(H,S\right)+C_{a}\left(H,S\right)\right) \end{array}$$

W(H,U)+rV(H,U)>W(H,D)+rV(H,D)

$$W_{h}+rV_{h}>0$$ (A.1)

R1: The receiver’s criteria, low quality signallers (node 11 vs. 12):

$$\begin{array}{l} q\left(W\left(L,U\right)+rV\left(L,U\right)\right)+\left(1-q\right)\left(W\left(L,D\right)+rV\left(L,D\right)\right)-C_{r}\left(SA\right)-r\left(C\left(L,N\right)+C_{a}\left(L,N\right)\right)<\\ q\left(W\left(L,D\right)+rV\left(L,D\right)\right)+\left(1-q\right)\left(W\left(L,D\right)+rV\left(L,D\right)\right)-C_{r}\left(SA\right)-r\left(C\left(L,N\right)+C_{a}\left(L,N\right)\right) \end{array}$$

W(L,U)+rV(L,U)<W(L,D)+rV(L,D)

$$W_{l}+rV_{l}<0$$ (A.2)

S2: The signaller’s criteria for high quality signallers (node 1 vs. 4):

$$\begin{array}{l} p\left(V\left(H,U\right)+rW\left(H,U\right)\right)+\left(1-p\right)\left(V\left(H,D\right)+rW\left(H,D\right)\right)-C\left(H,S\right)-C_{a}\left(H,S\right)-rC_{r}\left(SA\right)>\\ p\left(V\left(H,D\right)+rW\left(H,D\right)\right)+\left(1-p\right)\left(V\left(H,D\right)+rW\left(H,D\right)\right)-C\left(H,N\right)-C_{a}\left(H,S\right)-rC_{r}\left(SA\right) \end{array}$$

p(V(H,U)+rW(H,U))−C(H,S)>p(V(H,D)+rW(H,D))−C(H,N)

$$p\left(V_{h}+rW_{h}\right)>C_{h}$$ (A.3)

S2: The signaller’s criteria for low quality signallers (node 9 vs node 12):

$$\begin{array}{l} q\left(V\left(L,U\right)+rW\left(L,U\right)\right)+\left(1-q\right)\left(V\left(L,D\right)+rW\left(L,D\right)\right)-C\left(L,S\right)-C_{a}\left(L,N\right)-rC_{r}\left(SA\right)<\\ q\left(V\left(L,D\right)+rW\left(L,D\right)\right)+\left(1-q\right)\left(V\left(L,D\right)+rW\left(L,D\right)\right)-C\left(L,N\right)-C_{a}\left(L,N\right)-rC_{r}\left(SA\right) \end{array}$$

q(V(L,U)+rW(L,U))−C(L,S)<q(V(L,D)+rW(L,D))−C(L,N)

$$q\left(V_{l}+rW_{l}\right)<C_{l}$$ (A.4)

R1: searching stage for receivers, node 1 vs. 5 and 12 vs. 16 for high and low quality signallers respectively—weighted by the probabilities of high and low quality signallers:

$$\begin{array}{l} x\left(p\left(W\left(H,U\right)+rV\left(H,U\right)\right)+\left(1-p\right)\left(W\left(H,D\right)+rV\left(H,D\right)\right)-C_{r}\left(SA\right)-r\left(C\left(H,S\right)+C_{a}\left(H,S\right)\right)\right)+\\ \left(1-x\right)\left(q\left(W\left(L,D\right)+rV\left(L,D\right)\right)+\left(1-q\right)\left(W\left(L,D\right)+rV\left(L,D\right)\right)-C_{r}\left(SA\right)-r\left(C\left(L,N\right)+C_{a}\left(L,N\right)\right)\right)>\\ x\left(\alpha p\left(W\left(H,U\right)+rV\left(H,U\right)\right)+\left(1-\alpha p\right)\left(W\left(H,D\right)+rV\left(H,D\right)\right)-C_{r}\left(NS\right)-r\left(C\left(H,S\right)+C_{a}\left(H,S\right)\right)\right)+\\ \left(1-x\right)\left(W\left(L,D\right)+rV\left(L,D\right)-C_{r}\left(NS\right)-r\left(C\left(L,N\right)+C_{a}\left(L,N\right)\right)\right) \end{array}$$

$$\begin{array}{l} x\left(p\left(W\left(H,U\right)+rV\left(H,U\right)\right)+(1-p)\left(W\left(H,D\right)+rV\left(H,D\right)\right)\right)\\ +(1-x)\left(W\left(L,D\right)+rV\left(L,D\right)\right)-C_{r}(SA)\\ >x\left(\alpha p\left(W\left(H,U\right)+rV\left(H,U\right)\right)+\left(1-\alpha p\right)\left(W\left(H,D\right)+rV\left(H,D\right)\right)\right)\\ +(1-x)\left(W\left(L,D\right)+rV\left(L,D\right)\right)-C_{r}(NS) \end{array}$$

$$\begin{array}{l} x\left(p\left(W\left(H,U\right)+rV\left(H,U\right)\right)+(1-p)\left(W\left(H,D\right)+rV\left(H,D\right)\right)\right)-C_{r}(SA)\\ >x\left(\alpha p\left(W\left(H,U\right)+rV\left(H,U\right)\right)+\left(1-\alpha p\right)\left(W\left(H,D\right)+rV\left(H,D\right)\right)\right)-C_{r}(NS) \end{array}$$

$$x\left(p\left(1-\alpha \right)\left(W\left(H,U\right)+rV\left(H,U\right)\right)-p\left(1-\alpha \right)\left(W\left(H,D\right)+rV\left(H,D\right)\right)\right)>C_{r}$$

$$xp(1-\alpha )(W_{h}+rV_{h})>C_{r}$$ (A.5)

S1: searching stage for high quality signallers (node 1 vs. 9):

$$\begin{array}{l} p\left(V\left(H,U\right)+rW\left(H,U\right)\right)+\left(1-p\right)\left(V\left(H,D\right)+rW\left(H,D\right)\right)-C\left(H,S\right)-C_{a}\left(H,S\right)-rC_{r}\left(SA\right)>\\ q\left(V\left(H,U\right)+rW\left(H,U\right)\right)+\left(1-q\right)\left(V\left(H,D\right)+rW\left(H,D\right)\right)-C\left(H,S\right)-C_{a}\left(H,N\right)-rC_{r}\left(SA\right) \end{array}$$

$$(p-q)(V_{h}+rW_{h})>C_{ah}$$ (A.6)

S1: searching stage for low quality signallers (node 4 vs. 12):

$$\begin{array}{l} p\left(V\left(L,D\right)+rW\left(L,D\right)\right)+\left(1-p\right)\left(V\left(L,D\right)+rW\left(L,D\right)\right)-C\left(L,N\right)-C_{a}\left(L,S\right)-rC_{r}\left(SA\right)<\\ q\left(V\left(L,D\right)+rW\left(L,D\right)\right)+\left(1-q\right)\left(V\left(L,D\right)+rW\left(L,D\right)\right)-C\left(L,N\right)-C_{a}\left(L,N\right)-rC_{r}\left(SA\right) \end{array}$$

$$0<C_{al}$$ (A.7)

R2: The receiver’s criteria when it is worth to give the resource to low quality signallers (node 3 vs. 4):

$$\begin{array}{l} p\left(W\left(L,U\right)+rV\left(L,U\right)\right)+\left(1-p\right)\left(W\left(L,D\right)+rV\left(L,D\right)\right)-C_{r}\left(SA\right)-r\left(C\left(L,N\right)+C_{a}\left(L,S\right)\right)>\\ p\left(W\left(L,D\right)+rV\left(L,D\right)\right)+\left(1-p\right)\left(W\left(L,D\right)+rV\left(L,D\right)\right)-C_{r}\left(SA\right)-r\left(C\left(L,N\right)+C_{a}\left(L,S\right)\right) \end{array}$$

$$\begin{array}{l} p\left(W\left(L,U\right)+rV\left(L,U\right)\right)>\\ p\left(W\left(L,D\right)+rV\left(L,D\right)\right) \end{array}$$

$$W_{l}+rV_{l}>0$$ (A.8)

R1: searching stage for receivers when it is worth to give the resource to low quality signaller as well (node 1 vs 5 and node 3 vs 7 weighted by the freequency of high and low quality signallers respectively):

$$\begin{array}{l} x\left(p\left(W\left(H,U\right)+rV\left(H,U\right)\right)+\left(1-p\right)\left(W\left(H,D\right)+rV\left(H,D\right)\right)-C_{r}\left(SA\right)-r\left(C\left(H,S\right)+C_{a}\left(H,S\right)\right)\right)+\\ \left(1-x\right)\left(p\left(W\left(L,U\right)+rV\left(L,U\right)\right)+\left(1-p\right)\left(W\left(L,D\right)+rV\left(L,D\right)\right)-C_{r}\left(SA\right)-r\left(C\left(L,N\right)+C_{a}\left(L,S\right)\right)\right)>\\ x\left(\alpha p\left(W\left(H,U\right)+rV\left(H,U\right)\right)+\left(1-\alpha p\right)\left(W\left(H,D\right)+rV\left(H,D\right)\right)-C_{r}\left(NS\right)-r\left(C\left(H,S\right)+C_{a}\left(H,S\right)\right)\right)+\\ \left(1-x\right)\left(\alpha p\left(W\left(L,U\right)+rV\left(L,U\right)\right)+\left(1-\alpha p\right)\left(W\left(L,D\right)+rV\left(L,D\right)\right)-C_{r}\left(NS\right)-r\left(C\left(L,N\right)+C_{a}\left(L,S\right)\right)\right) \end{array}$$

$$\begin{array}{l} x\left(p\left(1-\alpha \right)\left(W\left(H,U\right)+rV\left(H,U\right)\right)-p\left(1-\alpha \right)\left(W\left(H,D\right)+rV\left(H,D\right)\right)\right)+\\ \left(1-x\right)\left(p\left(1-\alpha \right)\left(W\left(L,U\right)+rV\left(L,U\right)\right)-p\left(1-\alpha \right)\left(W\left(L,D\right)+rV\left(L,D\right)\right)\right)>\\ C_{r}\left(SA\right)-C_{r}\left(NS\right) \end{array}$$

$$p\left(1-\alpha \right)\left[x(W_{h}+rV_{h})+(1-x)(W_{l}+rV_{l})\right]>C_{r}$$ (A.9)

S1: searching stage for low quality signallers when it is worth to give the resource to low quality signaller as well (node 3 vs 11):

$$\begin{array}{l} p\left(V\left(L,U\right)+rW\left(L,U\right)\right)+\left(1-p\right)\left(V\left(L,D\right)+rW\left(L,D\right)\right)-C\left(L,N\right)-C_{a}\left(L,S\right)-rC_{r}\left(SA\right)>\\ q\left(V\left(L,U\right)+rW\left(L,U\right)\right)+\left(1-q\right)\left(V\left(L,D\right)+rW\left(L,D\right)\right)-C\left(L,N\right)-C_{a}\left(L,N\right)-rC_{r}\left(SA\right) \end{array}$$

$$\begin{array}{l} p\left(V\left(L,U\right)+rW\left(L,U\right)\right)-p\left(V\left(L,D\right)+rW\left(L,D\right)\right)-C_{a}\left(L,S\right)>\\ q\left(V\left(L,U\right)+rW\left(L,U\right)\right)-q\left(V\left(L,D\right)+rW\left(L,D\right)\right)-C_{a}\left(L,N\right) \end{array}$$

$$(p-q)(V_{l}+rW_{l})>C_{al}$$ (A.10)
